# Prescription Value-Based Automatic Optimization of Importance Factors in Inverse Planning

**DOI:** 10.1177/1533033819892259

**Published:** 2019-11-29

**Authors:** Caiping Guo, Pengcheng Zhang, Zhiguo Gui, Huazhong Shu, Lihong Zhai, Jinrong Xu

**Affiliations:** 1 Department of Electronic Engineering, Taiyuan Institute of Technology, Taiyuan, China; 2 Shanxi Provincial Key Laboratory for Biomedical Imaging and Big Data, North University of China, Taiyuan, China; 3 Laboratory of Image Science and Technology, Southeast University, Nanjing, China; 4 Centre de Recherche en Information Médicale Sino-français (CRIBs), Rennes, France

**Keywords:** importance factor, intensity-modulated radiation therapy, prescription value, automatic planning

## Abstract

**Objective:**

An automatic method for the optimization of importance factors was proposed to improve the efficiency of inverse planning.

**Methods:**

The automatic method consists of 3 steps: (1) First, the importance factors are automatically and iteratively adjusted based on our proposed penalty strategies. (2) Then, plan evaluation is performed to determine whether the obtained plan is acceptable. (3) If not, a higher penalty is assigned to the unsatisfied objective by multiplying it by a compensation coefficient. The optimization processes are performed alternately until an acceptable plan is obtained or the maximum iteration *N*_max_ of step (3) is reached.

**Results:**

Tested on 2 kinds of clinical cases and compared with manual method, the results showed that the quality of the proposed automatic plan was comparable to, or even better than, the manual plan in terms of the dose–volume histogram and dose distributions.

**Conclusions:**

The proposed algorithm has potential to significantly improve the efficiency of the existing manual adjustment methods for importance factors and contributes to the development of fully automated planning. Especially, the more the subobjective functions, the more obvious the advantage of our algorithm.

## Introduction

Inverse planning in intensity-modulated radiotherapy (IMRT) aims to deliver uniform doses to the planning target volume (PTV), while sparing damage to normal tissues (NTs) and organs at risk (OARs). The performance of an inverse planning system depends on the optimization engine, which handles mutually exclusive optimization goals for different structures and accordingly adjusts beamlet intensities using an iterative method.^1^ In standard inverse planning, such a trade-off is commonly resolved by minimizing a therapeutic objective function, which combines different structure-specific penalty objectives using importance factors. Two methods are commonly used to adjust the importance factors (weighting factors): fine-tuning importance factors in a manual trial-and-error fashion^2,3^ and multiobjective optimization, where part of the representatives of a Pareto surface are precomputed and then navigated.^1,4-9^ Between the 2 methods, the former is the most commonly used, although it is time-consuming, because lengthy manual trial-and-error procedures are needed to find a set of suitable importance factors to arrive at a satisfactory balance between the PTV coverage and OAR sparing. Moreover, the optimization process becomes even more complicated and time-consuming as the number of subobjective functions increases. To improve the efficiency of inverse planning, it is desirable to have an automatic, or a more effective, approach to determine the importance factors.

To achieve this goal, some authors have proposed schemes to automatically adjust the importance factors. Xing *et al* ^10^ proposed a method for auto-optimizing importance factors using 2-stage optimization under the guidance of a predefined dose–volume histogram (DVH) score function. In a similar vein, some researchers proposed methods to automatically optimize treatment plans guided by a reference plan.^8,11-24^ Of these, the source of the reference plan, for different clinical applications, is not the same. For automatic replanning, the reference plan comes from a clinically delivered original plan for the same patient. For knowledge-based approaches, the reference plan for an incoming cancer patient is based on retrospective patient data. These methods can automatically adjust the importance factors but require a prechosen DVH curve for each optimized organ. Due to the differences and variances of patient-specific anatomical structures, it is difficult to choose beforehand the ideal DVH curves for both the traditional inverse planning and replanning process. Additionally, some investigators, using machine learning, have proposed methods to predict OAR weights^25^ and OAR DVHs^26-28^ for some cancer cases. Dias *et al* ^29^ implemented the automatic optimization of prescribed doses and importance factors by applying the theory of fuzzy inference. All the aforementioned methods are based on dose-based optimization models.

In this study, we focused on automatic optimization for IMRT treatment planning to avoid the requirement of human planner intervention. An effective and simple computer-aided method to automatically and iteratively adjust the importance factors without manual intervention was proposed. Moreover, compared with the aforementioned automatic important factor optimization methods, the advantages of our proposed method are: (1) It does not need a reference plan, but is suitable for automatic optimization method based on a reference plan; (2) It does not need a large number of clinical cases to train prediction model; (3) It does not need to design complex membership functions; and (4) It not only applies to dose-based physical optimization, but also to others, such as biological and hybrid optimization.

## Materials and Methods

### Optimization Technique

**Figure 1.**
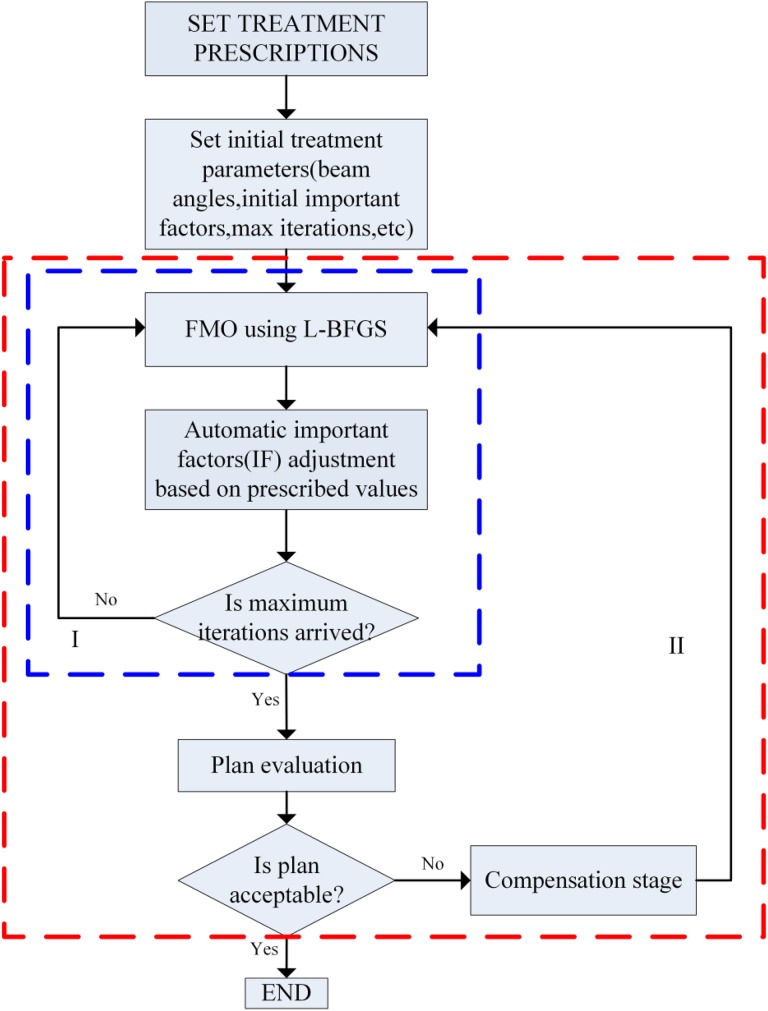
Overview of the automatic FMO. FMO indicates fluence map optimization.

The core of the automatic method is the iterative and automatic adjustment of importance factors based on the difference between actual dose and the prescribed value within a certain small number of iterations. Figure 1 depicts a flow chart of the automatic method. (1) First, the importance factors are automatically and iteratively adjusted based on proposed penalty strategies. (2) Then, plan evaluation is performed to determine whether the obtained plan is acceptable according to evaluation functions. (3) If not, a higher penalty is assigned to the unsatisfied objective by multiplying a compensation coefficient. The optimization processes are performed iteratively until an acceptable plan is obtained or the maximum iteration *N*_max_ of step (3) is reached. These optimization problems were solved using the limited-memory Broyden–Fletcher–Goldfarb–Shanno gradient optimization algorithm,^30^ and square roots of beamlet weights were used as variables to avoid nonphysical solutions with negative values.^1^ The following sections provide details of the automatic method.

#### Prescription value-based penalty strategies

In clinical practice, prescription values mainly include minimum dose, maximum dose, mean dose, maximum dose–volume (DV) constraints, minimum DV constraints, generalized equivalent uniform dose (gEUD),^31^ tumor control probability (TCP), and NT complication probability (NTCP).^32^ We use $w_{\text{new}}$ and $w_{\text{old}}$ to, respectively, denote the current importance factors and automatically adjusted importance factors, where we defined the relationship between them as

1 $$w_{\text{new}}=w_{\text{old}}+\text{factor}.$$

where factor is defined as weight correction factor which is a function of prescription value and actual value. For different prescription values, the factor proposed in this article is defined in Table 1. The subscripts pre and cur represent the prescription value and the actual value in the current plan, respectively. For DV constraint, *V*_1_ is the prescribed volume with respect to prescribed dose *D*_1_ and *V*_2_ the actual volume in the given plan with respect to *D*_1_.

**Table 1.** Weight Correction Factors for Different Prescription Values.

Prescription Value	Factor
$D_{\text{min-pre}}$	$D_{\text{min-pre}}/D_{\text{min}}{\;}_{\text{-cur}}$
$D_{\text{max-pre}}$	$D_{\text{max-cur}}\text{/}D_{\text{max-pre}}$
$D_{\text{mean-pre}}$	$\text{max(}D_{\text{mean-cur}}\text{,}D_{\text{mean-pre}}\text{)/min(}D_{\text{mean-cur}}\text{,}D_{\text{mean-pre}}\text{)}$ for PTV
$D_{\text{mean-pre}}$	$D_{\text{mean-cur}}\text{/}D_{\text{mean-pre}}$ for OAR
$DV_{\text{max}}$	$V_{2}/V_{1}$
$DV_{\text{min}}$	$V_{\text{1}}/V_{\text{2}}$
gEUD	${\text{gEUD}}_{\text{cur}}{\text{/gEUD}}_{\text{pre}}\text{,}\;a\geq \text{1}$ for OAR
gEUD	${\text{gEUD}}_{\text{pre}}{\text{/gEUD}}_{\text{cur}},a<\text{1}$ for PTV
NTCP	${\text{NTCP}}_{\text{cur}}{\text{/NTCP}}_{\text{pre}}$
TCP	${\text{TCP}}_{\text{pre}}{\text{/TCP}}_{\text{cur}}$

Abbreviations: gEUD, generalized equivalent uniform dose; NTCP, normal tissue complication probability; OAR, organ at risk; PTV, planning target volume; TCP, tumor control probability.

For example, the minimum dose criterion is typically used to control the low dose delivered to PTV. The penalty for it is the greater the difference between $D_{\text{min}}{\;}_{\text{-cur}}$ and $D_{\text{min-pre}}$, the greater the corrector factor. Hence, a higher importance factor is assigned to the minimum-dose-based subscore. But maximum dose criterion is typically used to constrain the overdose delivered to PTV or OAR. The greater the maximum dose of the current plan, the greater the factor. Other penalty strategies are formatted similarly.

The automatic adjustment of importance factors, shown in rectangle I in Figure 1, compromises the requirement of PTV coverage and OARs sparing based on prescription value-based penalty strategies. Then, plan evaluation is performed.

#### Plan evaluation

Plan evaluation is performed according to 2 evaluation functions, which are provided in Equations 2 and 3, which are respectively express the PTV coverage considered in priority, and the maximum DV objectives for OARs. *N* is the number of the DV constraints.

2 $$f_{\text{1}}=V_{\text{PTV}}\text{(}D_{\text{min}\;\text{pre}}\text{)}\geq \text{95\%}$$

3 $$f_{\text{2}}=V_{\text{OAR}}(D_{\text{1}i})\leq V_{\text{1}i}\text{\%}\;i=\text{1}\cdots N$$

The criteria for plan evaluation are listed as follows: (1) If the PTV coverage is satisfied, while the specific DV constraints of OARs are not satisfied, the algorithm considers improving the OARs DV constraints without jeopardizing the prescribed PTV coverage. If the OAR doses cannot be made to satisfy all the conditions without compromising the prescribed PTV coverage, the high-dose region of the OAR will be considered first. (2) If the PTV coverage is dissatisfied, a higher penalty, imposed by multiplying by a compensation coefficient, is assigned to the weight correction factors of the subscores controlling PTV, until the dose constraint of the PTV was satisfied. (3) If the PTV coverage and OARs DV constraints are all satisfied, the algorithm tries to improve the PTV coverage as far as possible while ensuring the prescribed OARs DV constraints; if OAR doses are needed to decrease to the fullest extent, the algorithm can try to decrease the OAR doses as far as possible while ensuring the prescribed PTV coverage.

Ideally, if the prescription values are well-defined, an acceptable plan is generated only by the step in Figure 1 (rectangle I). Otherwise, a compensation stage will be performed.

#### Compensation stage

**Figure 2.**
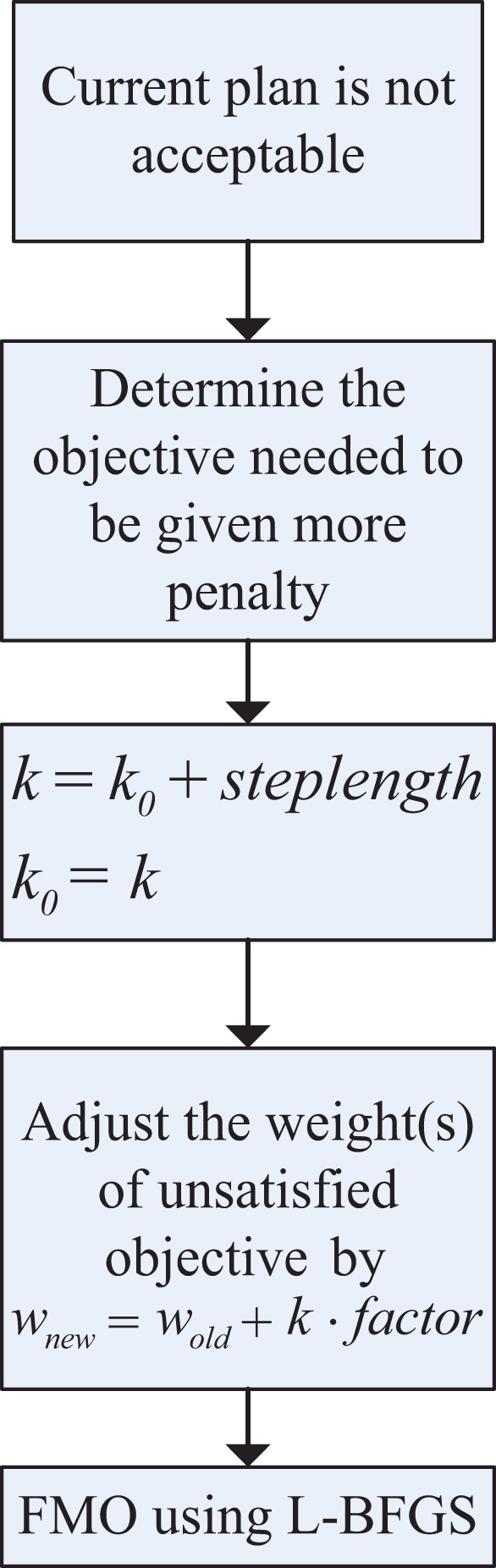
Process of compensation stage.

During the compensation stage, the unsatisfied objective is assigned a higher penalty by multiplying by a coefficient *k* defined in Equation 4. Then, the fluence map optimization (FMO) shown in rectangle I in Figure 1 is performed once again. Figure 2 shows the process, $k_{\text{0}}$ represents the initial value, and steplength represents the variation of the *k* between iterations.

4 $$w_{\text{new}}=w_{\text{old}}+k\cdot \text{factor}$$

#### Objective function

Three representative objective functions (DV-based objective function, gEUD-based physical–biological objective function, and NTCP-based physical–biological objective function) were used in our study. The weighted sum of objective function *f* to be minimized is

5 $$f\text{(}D\text{)}=\sum_{i=\text{1}}^{l}w_{i}f_{i}\text{(}D\text{)}\text{,}$$

where importance factor *w_i_* represents clinical significance, *l* is the number of subobjective functions, and ***D*** is the dose distributions to the organ constrained by subscore *f_i_*. To avoid numerical errors in the automatic procedure, the importance factors were kept within a specific range through normalization by Equation 6. The importance factors were kept within (0, 1). It is worth noting that the normalization does not affect the optimal solution from the mathematical standpoint.

6 $$w_{i}=\frac{w_{i}}{\sum_{i=\text{1}}^{l}w_{i}}$$

#### DV-based objective function

Dose–volume-based optimization is common in clinical settings for IMRT treatment planning. The DV-based model used in our study was

7 $$f\text{(}D\text{(x))}=w_{\text{1}}f_{\text{Min}}\text{(}D_{\text{PTV}}\text{)}+w_{\text{2}}f_{\text{Mean}}\text{(}D_{\text{PTV}}\text{)}\;+\sum_{i=\text{3}}^{\text{5}}w_{i}f_{{\text{DV}}_{\text{max}}-\text{OAR1}}\text{(}D\text{)}+\sum_{i=\text{6}}^{\text{8}}w_{i}f_{DV_{\text{max}}-\text{OAR2}}\text{(}D\text{)}+\cdots$$

Due to the nonconvexity of the DV criterion,^33^ the equivalent convex DV criterion was used to construct the maximum DV subscore.^34^ The subscores used in DV-based optimization model are expressed as follows:

8 $$f_{Min}\text{(}D\text{)}=\frac{\text{1}}{N}\sum_{i=\text{1}}^{N}H\text{(}D_{Min}-D_{i}\text{)}\cdot {\text{(}D_{Min}-D_{i}\text{)}}^{\text{2}}\text{,}$$

9 $$f_{\text{Mean}}\text{(}D\text{)}=\frac{\text{1}}{N}\sum_{i=\text{1}}^{N}{\text{(}D_{i}-D_{\text{Mean}}\text{)}}^{\text{2}}\;\;\text{,}$$

10 $$f_{DV_{\text{max}}}\text{(}D\text{)}=\frac{1}{N}\sum_{i=\text{1}}^{N}H\text{(}D_{i}-D_{\text{1}}\text{)}\cdot H\text{(}D_{2}-D_{i}\text{)}\cdot {\text{(}D_{i}-D_{\text{1}}\text{)}}^{\text{2}}\;\text{,}$$

where *H*(*x*) represents the step function; *D*_i_ is the dose in the *i*th voxel; *N* is the number of voxels in PTV or OAR; the maximum DV constraint $V_{D_{\text{1}}}\leq V_{\text{1}}\text{\%}$ indicates that the volume of the OAR receiving dose greater than *D*_1_ should be less than $V_{\text{1}}\text{\%}$, *D*_2_ is in the current plan, where $V_{D_{\text{2}}}=V_{\text{1}}\text{\%}$.

#### gEUD-based physical–biological objective function

The advantages of gEUD-based optimization have been widely investigated.^3,6,34-41^ Previous studies have shown the superiority of gEUD-based physical–biological optimization compared with gEUD-based biological optimization.^37,40^ Based on their findings, the gEUD-based objective function used in our work was,

11 $$\begin{array}{l} f\text{(}D\text{)}=w_{\text{1}}f_{\text{Min}}\text{(}D_{\text{PTV}}\text{)}+w_{\text{2}}f_{\text{Mean}}\text{(}D_{\text{PTV}}\text{)}+\;\sum_{i=\text{1}}^{N_{\text{OAR}}}w_{i+\text{2}}f_{\text{gEUD}}\text{(}D\text{),} \end{array}$$

where

12 $$f_{\text{gEUD}}\text{(}D\text{)}=H{\text{(gEUD}}_{\text{cur}}\text{(}D\text{)}-{\text{gEUD}}_{\text{pre}}\text{)}\cdot {\text{(gEUD}}_{\text{cur}}\text{(}D\text{)}-{\text{gEUD}}_{\text{pre}}\text{),}$$

$N_{\text{OAR}}$ is the number of OARs; gEUD is given by Niemieko,^31^ the *w_i_* is given from Equation 6.

#### NTCP-based physical–biological objective function

NTCP-based optimization has been investigated in inverse treatment planning^42-46^ and incorporated into some commercial treatment planning software.^44,47^ The NTCP-based objective function was expressed in Equation 13. The NTCP model used here was the LKB model.^48,49^

13 $$f\text{(}D\text{)}=w_{\text{1}}f_{\text{Min}}\text{(}D_{\text{PTV}}\text{)}+w_{\text{2}}f_{\text{Mean}}\text{(}D_{\text{PTV}}\text{)}+\sum_{i=\text{1}}^{N_{\text{OAR}}}\omega_{i+\text{2}}f_{\text{NTCP}}\text{(}D\text{)}.$$

To use a gradient-based optimization algorithm to solve the nonconvex NTCP-based optimization problem, the following equivalent convex NTCP criterion^38^ was applied. The NTCP-based subscore^50^ utilized in our work was defined in Equation.^14^

14 $$f_{\text{NTCP}}\text{(}D\text{)}=\text{max(ln(1}-{\text{NTCP}}_{\text{pre}}\text{)}-\text{ln(1}-{\text{NTCP}}_{\text{LKB}}\text{(}D\text{)},0\text{)}$$

### Patients, Related Parameters, and Assessment Criteria

The feasibility and performance of the automatic method was tested on 10 cases of prostate cancer and 3 cases of head and neck (HN) cancer randomly selected from the database of treated cases. The study protocols were approved by the Ethics Committee of the North University of China with the approval No. 2018006, the written consent forms were signed by the participants whose computed tomography (CT) images were used for this study.

For prostate cancer cases, these patients underwent simulation and treatment in the supine position. Target volume and OARs (bladder, rectum, and femoral heads) were delineated on CT slices. The rectum and bladder walls were generated with a thickness of 5 mm from the external manually delineated rectal and bladder contours, respectively. A “tissue ring,” the outside of the area extending the PTV by 5 cm, was defined as the other NT. The target volume included the prostate and excluded the pelvic lymph nodes. The PTV was calculated by adding a 10 mm margin in all directions except the posterior, where a 5 mm margin was applied. All plans used the identical configuration of 5 coplanar 6 MV photon beams, with gantry angles of 36°, 100°, 180°, 260°, and 324°. The values of *D*_1_ and *V*_1_ for the DV-based optimization are listed in Table 2. The prescribed *D*_min-pre_ and *D*_mean-pre_ for PTV were 74 Gy and 78 Gy, respectively. The prescribed ${\text{gEUD}}_{\text{pre}}$ and ${\text{NTCP}}_{\text{pre}}$ for rectum and bladder were 60 Gy and 0.05, respectively. The NTCP and gEUD radiobiological parameters for the bladder and rectum were taken from studies, respectively.^51,52^

**Table 2.** *D*_1_ and *V*_1_ for the DV Subobjective Functions.

OAR	*D*_1_ (Gy)	*V*_1_ (%)
Rectum	50	40
	65	25
	75	15
Bladder	65	35
	70	30
	75	16

Abbreviations: DV, dose–volume; OAR, organ at risk.

For HN cancer cases, patients, related optimization parameters applied in Equations 7 and 11, and plan assessment criteria are same as those in our previous study.^53^ The prescribed ${\text{NTCP}}_{\text{pre}}$ for OARs (spinal cord, brainstem, L-parotid, and R-parotid) was 0.1.

The number of maximum iterations was 5 in rectangle I in Figure 1, empirically chosen through a series of experiments. The dose sedimentary matrix was calculated with a standard pencil beam algorithm,^54^ implemented on the computational environment for radiotherapy research (CERR).^55^ All experiments were performed by using an instrument equipped with a 32-bit OS, Windows 7, and an Intel (R) Core (TM) i3-4150 CPU with 4G RAM.

The plan quality was assessed by using the clinical evaluation guidelines shown in Table 3.^56^ Conformity index (CI) and homogeneity index (HI)^57^ are, respectively, defined in Equations 15 and 16.

15 $$\text{CI=}\frac{V_{\text{τ,ref}}}{V_{\text{τ}}}\text{×}\frac{V_{\text{τ,ref}}}{V_{\text{ref}}}\text{,}$$

where $V_{\text{τ}}$ is the volume of the PTV, $V_{\text{τ,ref}}$ is the target volume that receives a dose greater than or equal to the reference (prescribed) dose, and $V_{\text{ref}}$ is the total volume that receives a dose greater than or equal to the reference dose.

16 $$\text{HI=}\frac{D_{\text{5\%}}}{D_{\text{95\%}}}\text{,}$$

where *D*_5%_ and *D*_95%_ correspond to the minimum doses delivered to the hottest 5% and 95% of the PTV, respectively.

All statistical tests were performed using the Wilcoxon matched-pair, signed-rank test using a significance level of 0.05.

It should be pointed out that the differences of the DVH criteria for the bladder and rectum in Tables 2 and 3 are that the DVH criteria in Table 2 are prescribed DV constraints in Equation 10, while the DVH criteria in Table 3 are evaluation guidelines to determine whether optimized plan is clinically accepted. For different prostate patients, the evaluation guidelines are same, but the prescribed DV constraints should be tightened and relaxed depending on the patients’ different structures.

**Table 3.** Dose–Volume Criteria for Bladder and Rectum.

OAR	Parameters of DV constraints					
Bladder			*V*_65_ < 50%	*V*_70_ < 35%	*V*_75_ < 25%	*V*_80_ < 15%
Rectum	*V*_50_ < 50%	*V*_60_ < 35%	*V*_65_ < 25%	*V*_70_ < 20%	*V*_75_ < 15%	

Abbreviations: DV, dose–volume; OAR, organ at risk.

## Results

In Optimization Technique section, we proposed an automatic algorithm for dynamically generated acceptable importance factors for IMRT inverse treatment planning. Next, we describes an investigation of the automatic method.

### Determination of Iteration Number

First, we investigated the impact of iteration numbers on the convergence of the algorithm and plan quality. It was found that the total iteration number to be 15, which is always enough to get an acceptable plan based on our a series of experiments. Then by using different combinations of iteration numbers of iterative adjustment shown in rectangle I in Figure 1 and compensation stage shown in rectangle II in Figure 1, we found that by performing compensation stage, the algorithm can further improve the plan quality than only by performing iterative adjustment shown in rectangle I in Figure 1. But after each adjustment of the compensation coefficient, because of the repetitive performing of iterative adjustment shown in rectangle I in Figure 1, the computation time becomes longer. As this test was consistent for different cases, we fixed the number of iterations at 5 in rectangle I in Figure 1 and the maximum iteration was 10 in rectangle II in Figure 1, which allows the algorithm to show the trade-off between the gain in plan quality and the cost in computation time.

### Effects of Compensation Coefficient

Under the above-prescription values, Table 4 lists the optimized compensation coefficient *k* for testing cases in 3 kinds of optimization methods. It was observed that in the DV-based optimization and gEUD-based optimization, the weight correction factors of subscores for PTV needed to be given extra penalty by multiplying a compensation coefficient, whereas in the NTCP-based optimization, the subscore for rectum or parotids needed to be given extra penalty; *k*_0_ for the DV-based, gEUD-based, and NTCP-based optimizations were, respectively, 1, 10, and 1. The respective step lengths were 1, 5, and 1.

**Table 4.** Optimized Compensation Coefficient *k* for 3 Optimization Methods.

Case	DV-Based	gEUD-Based	NTCP-Based
	$w_{{\;}_{i\text{new}}}^{\text{PTV}}=w_{i\text{old}}^{\text{PTV}}$ +k⋅factor *i* = 1, 2 in Equation 7	$w_{{\;}_{i\text{new}}}^{\text{PTV}}=w_{i\text{old}}^{\text{PTV}}+k\cdot \text{factor}$ *i* = 1, 2 in Equation 11	$w_{i\text{new}}=w_{i\text{old}}+k\cdot \text{factor for rectum}$ and parotids *i* for rectum
Prostate 1	1	10	4
Prostate 2	10	30	1
Prostate 3	1	10	2
Prostate 4	8	20	1
Prostate 5	9	20	1
Prostate 6	3	20	4
Prostate 7	1	25	1
Prostate 8	1	25	1
Prostate 9	8	20	4
Prostate 10	10	15	2
HN 1	5	10	2
HN 2	2	20	1
HN 3	8	25	3

Abbreviations: DV, dose–volume; gEUD, generalized equivalent uniform dose; HN, head and neck; NTCP, normal tissue complication probability.

**Figure 3.**
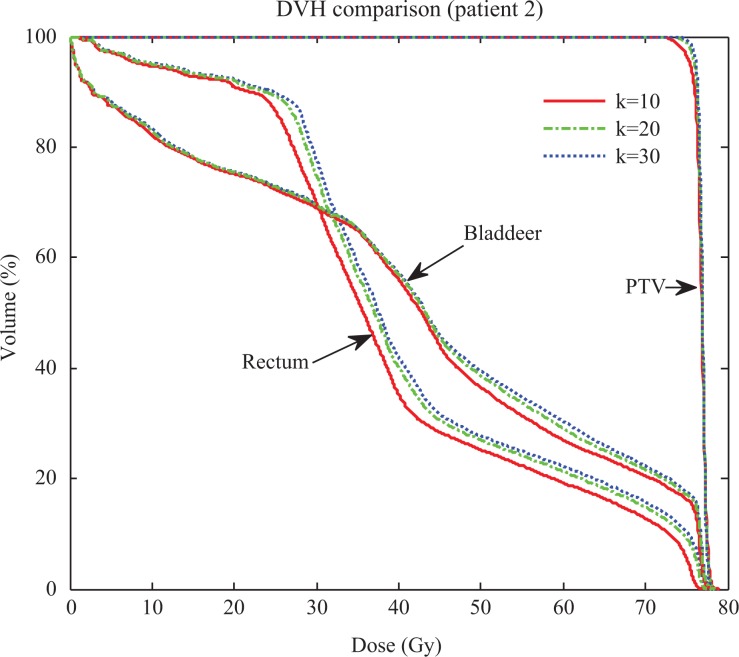
Dose–volume histograms of gEUD plans with different coefficient *k* for patient 2. gEUD indicates generalized equivalent uniform dose.

Figure 3 shows the evolutionary process of the gEUD-based optimization with different compensation coefficient *k* for prostate patient 2. It can be seen that the greater the important factors with higher coefficient *k* for the PTV subscores, the greater the dose coverage within the target, whereas OARs sparing decreased because of the trade-off.

**Figure 4.**
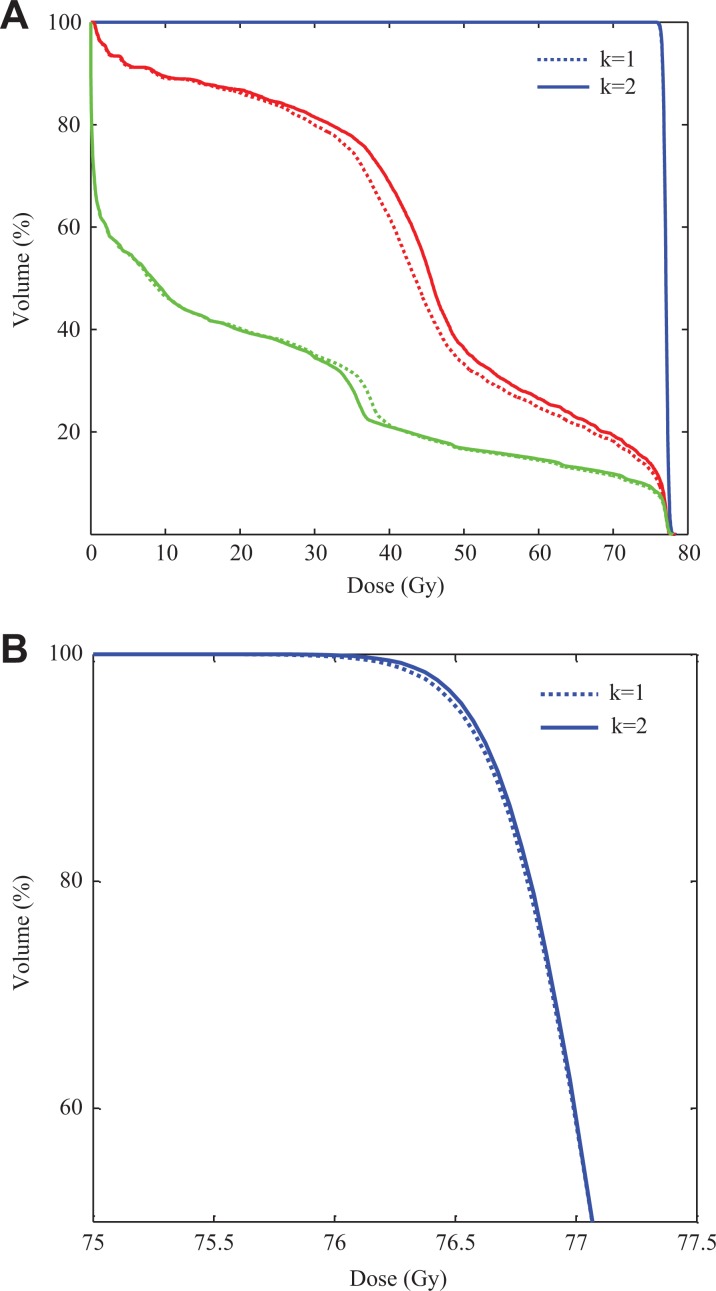
Dose–volume histograms of the NTCP plan with *k* = 1 and *k* = 2 for patient 8. A, full DVH curves; (B) high-dose region of the DVH curves. Blue curves for PTV, red curves for rectum, and green curves for bladder. DVH indicates dose–volume histogram; NTCP, normal tissue complication probability; PTV, planning target volume.

If we want to further improve the quality of the automatic plan for a specific objective, we can manually impose a compensation coefficient on the objective. Taking the optimized DV plan of prostate patient 8 for example, Figure 4 shows the difference between the plans with *k* = 1 and *k* = 2. The aim is to further improve the dose distribution of PTV with *k* = 2 yielding improved PTV coverage. The same approach can be used to improve other objectives.

### Plan Comparison

For the automatic plans, PTV coverage quantified by CI and HI is guaranteed for testing cases in 3 kinds of optimization methods (Table 5). It is clear that the proposed automatic method yields good dose conformity and homogeneity to the PTV.

**Table 5.** Dose Conformity and Homogeneity to the PTV of the Automatic Plan for 10 Prostate Cases.

Index	Method	Prostate										HN (PTV70, PTV63, PTV56)		
		1	2	3	4	5	6	7	8	9	10	1	2	3
	DV-based	0.88	0.87	0.90	0.88	0.90	0.85	0.85	0.85	0.86	0.86	0.84 0.59 0.48	0.89 0.53 0.36	0.80 0.45 0.25
CI	gEUD-based	0.88	0.87	0.90	0.87	0.90	0.86	0.85	0.86	0.85	0.86	0.83 0.60 0.49	0.86 0.50 0.35	0.78 0.44 0.29
	NTCP-based	0.88	0.87	0.90	0.87	0.89	0.85	0.85	0.84	0.87	0.86	0.84 0.67 0.41	0.88 0.51 0.36	0.78 0.42 0.27
	DV-based	1.02	1.02	1.04	1.02	1.06	1.03	1.02	1.02	1.05	1.02	1.04 1.08 1.06	1.07 1.09 1.05	1.05 1.10 1.10
HI	gEUD-based	1.02	1.02	1.04	1.02	1.06	1.04	1.02	1.02	1.04	1.02	1.08 1.10 1.08	1.09 1.10 1.06	1.05 1.13 1.10
	NTCP-based	1.02	1.02	1.04	1.02	1.06	1.02	1.02	1.01	1.02	1.02	1.07 1.09 1.06	1.08 1.13 1.08	1.07 1.15 1.11

Abbreviations: CI, conformity index; DV, dose–volume; gEUD, generalized equivalent uniform dose; HI, homogeneity index; NTCP, normal tissue complication probability; PTV, planning target volume.

**Figure 5.**
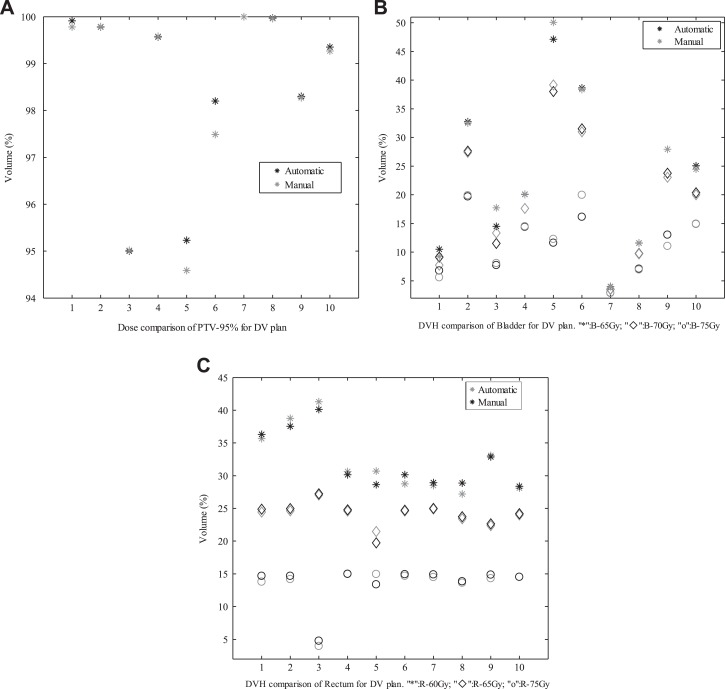
Comparisons of DV criteria for DV plan. DV indicates dose–volume. A, Dose comparison of PTV-95% for DV plan; (B) DVH comparison of Bladder for DV plan(“*”:B-65Gy,“♦”: B-70Gy,“o”: B-75Gy); (C) DVH comparison of Rectum for DV plan(“*”:R-60Gy,“♦”: R-65Gy,“o”: R-75Gy).

**Figure 6. fig6-1533033819892259:**
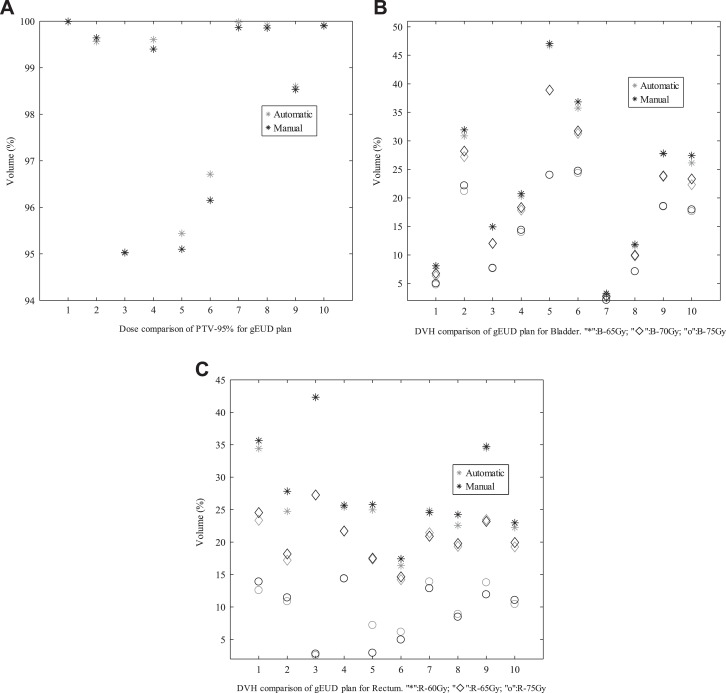
Comparisons of DV criteria for gEUD plan. DV indicates dose–volume; gEUD, generalized equivalent uniform dose. A, Dose comparison of PTV-95% for gEUD plan; (B) DVH comparison of Bladder for gEUD plan(“*”:B-65Gy,“♦”: B-70Gy,“o”: B-75Gy); (C) DVH comparison of Rectum for gEUD plan(“*”:R-60Gy,“♦”: R-65Gy,“o”: R-75Gy).

Next, the automatic plan was compared with the manual plan generated in CERR by experienced physicians and reported in other literature created by our team members.^50^ For prostate cancer cases, Figures 5 [Fig fig6-1533033819892259]–7 present the comparative results corresponding to DVH criteria for the PTV and OARs. The data labeled PTV-95% represent the volume fraction of the PTV receiving 0.95 × 78 Gy of radiation. The other labels are formatted similarly, where the acronyms correspond to the bladder (B) and rectum (R). The black scatter point represents the result of the automatic plan, and the grey scatter point represents the result of the manual plan. The comparative results demonstrate that the automatic method can generated clinically acceptable plans in terms of PTV coverage and OARs sparing. Moreover, the comparisons in Figures 5 [Fig fig6-1533033819892259]–7 indicate that the quality of the automatic plan is better than that of manual plan considering DV constraints. For example, the automatic DV plan for patient 5 in Figure 5 yields better PTV coverage and better trade-off between bladder-sparing and rectum-sparing compared to manual DV plan. The same conclusion can be applied to the other plan comparison for all testing cases.

**Figure 7.**
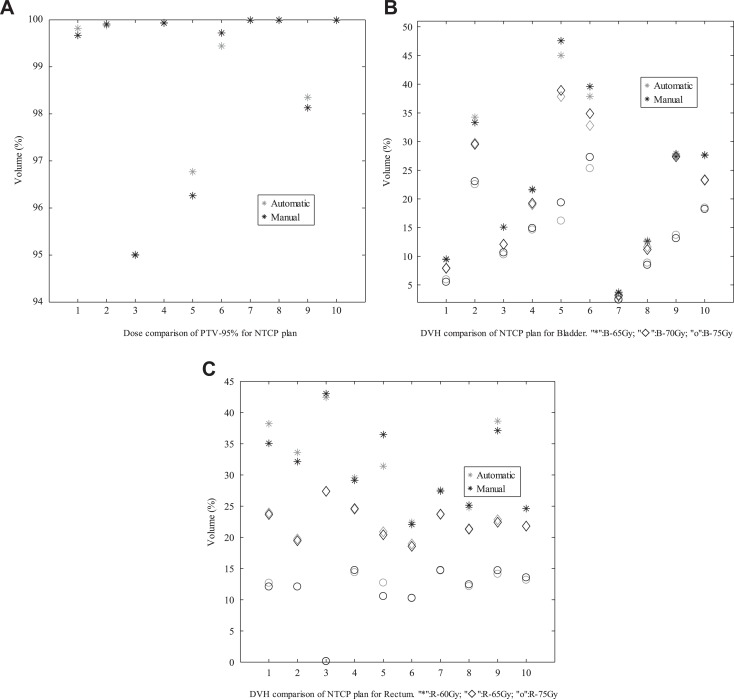
Comparisons of DV criteria for NTCP plan. DV indicates dose–volume; NTCP, normal tissue complication probability. A, Dose comparison of PTV-95% for NTCP plan; (B) DVH comparison of Bladder for NTCP plan(“*”:B-65Gy,“♦”: B-70Gy,“o”: B-75Gy); (C) DVH comparison of Rectum for NTCP plan(“*”:R-60Gy,“♦”: R-65Gy,“o”: R-75Gy).

**Figure 8.**
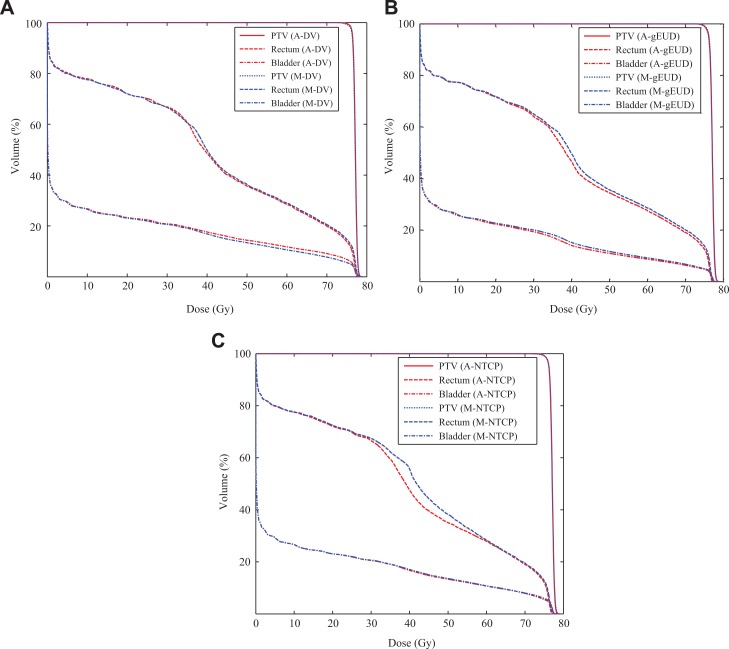
Dose–volume histograms for patient 1. A, Comparison between manual DV plan (M-DV) and automatic DV plan (A-DV); (B) comparison between manual gEUD plan (M-gEUD) and automatic gEUD plan (A-gEUD); (C) comparison between manual NTCP plan (M-NTCP) and automatic NTCP plan (A-NTCP). DV indicates dose–volume; gEUD, generalized equivalent uniform dose; NTCP, normal tissue complication probability.

Figure 8A-C present the comparative DVHs between the automatic plans and the manual plans based on DV model, gEUD model, and NTCP model for patient 1. The red curves present the automatic plan, and the blue curves present the manual plan. The acronyms “A” and “M” correspond to the automatic method and the manual method. The comparison of DVHs demonstrates that our proposed automatic method can result in better trade-off between PTV coverage and OARs sparing.

**Figure 9.**
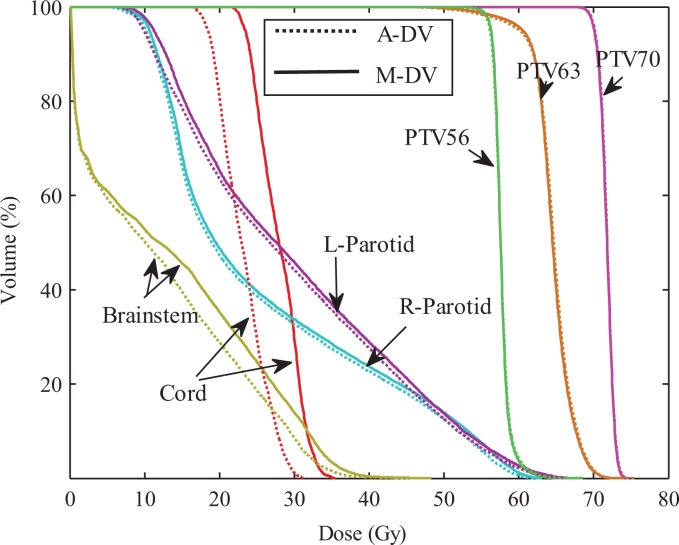
Average DVH comparisons of treatment plans. Dotted line: automatic DV plan; Solid line: manual DV plan. DV indicates dose–volume; DVH, dose–volume histogram.

For HN cases, the same comparisons were performed. Figure 9 compares the average DVHs for the automatic DV plan and the DV manual plan. It clearly shows improvement for all OARs. Similarly, comparisons corresponding to DVH criteria for the PTVs and OARs were also performed. The comparative results indicate that the automatic plans are better than that of manual plan in terms of OARs protection.

To further prove the efficiency of our proposed automatic method, we also considered spatial dose information. Consistent with the comparison of the DVHs, the automatic plans retained similar or better dose distributions compared to the manual plan.

### Statistical Analysis of Experimental Results

To compare the results between the automatic plan and the manual plan for all testing cases, the statistically analysis were performed. There are no significant differences in CI and HI that indicate the similar dose coverage to the PTVs between 2 kinds of plans. As shown in Figures 5 [Fig fig6-1533033819892259]–7, for 10 prostate cancer cases, no significant difference was found between these DV values for automatic plan and manual plan in 3 kinds of optimization methods. For 3 cases with HN cancer, the Wilcoxon test was performed for the difference dose bins of DVH in Figure 9, significant differences were observed for the *D*_mean_ to the cord and the brainstem.

### Changes of Importance Factors

**Figure 10.**
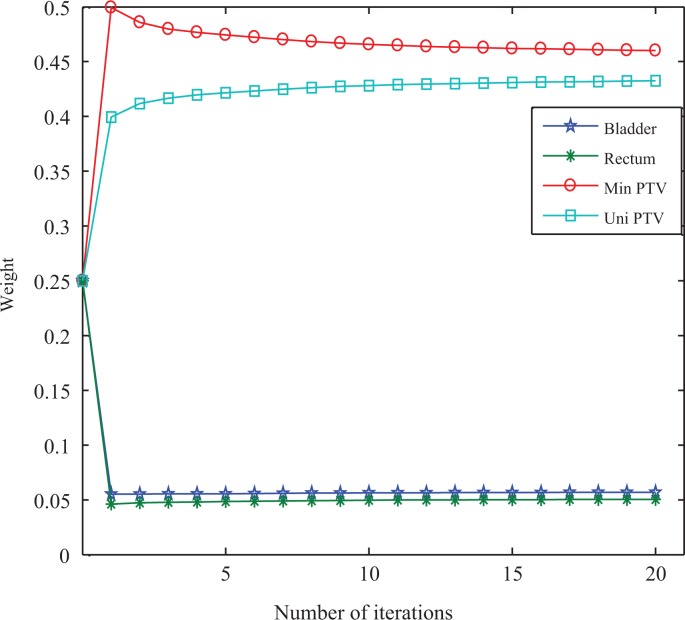
Variances of importance factors for patient 6 with prostate cancer in gEUD-based automatic optimization method. gEUD indicates generalized equivalent uniform dose.

To illustrate the variances of the importance factors in automatic method, taking gEUD-based optimization as an example for patient 6 with prostate cancer, Figure 10 shows the variances with respect to iterations. In the optimization of all other cases, the weight changes were similar.

### Influence of Initial Importance Factors

The proposed algorithm is intended to be automatic optimization for importance factors, so we would not like to have the planner initializing the importance factors by trial and error. In our experiments, we used uniform initial importance factors. For example, the uniform initial weights (1/4, 1/4, 1/4, 1/4) were used in gEUD-based optimization. We then tested the stability performance of the automatic method using different sets of initial importance factors generated randomly and found that different initial importance factors will only have as a consequence a decrease or an increase in the total computation time and not in the quality of the final plan.

### Influence of Prescribed Value

A series of experiments showed that whether the compensation step was needed correlated to the prescribed value. The value of the compensation coefficient *k* was different when optimization was guided by different prescribed values for the same case. This contributed to the fact that different prescribed values led to different penalties. We found that for the testing cases, the choice of prescription value only resulted in different values of *k* and not in the quality of the optimized treatment plan. Still, it should be pointed out that if the prescription values cannot result in acceptable plan within limited iterations, physicians manually adjust them according to their experience, and then repeat the automatic method.

## Discussions and Conclusions

In this article, we proposed a prescription value-based automatic importance factor optimization algorithm that avoids tedious manual trial-and-error schemes of the traditional manual method. Furthermore, this advantage becomes more and more apparent with increase in the number of subobjective functions. It should be pointed out that our proposed prescription value-based penalties strategies shown in Table 1 are novel and the compensation coefficient introduced in our algorithm can improve the flexibility of the new automatic method. Moreover, different initial importance factors have minimal impact on the plan quality. In our experiments, for different prostate cases and HN cases, it takes 3 to 7 minutes or 5 to 11 minutes to produce an acceptable plan applying our proposed automatic optimization method of importance factors, while for manual trial-and-error method, it needs experienced physicians to take 1 to 3 hours to obtained an acceptable plan. The substantial reduction in human intervention will greatly improve the efficiency of radiotherapy. The automatic optimization of importance factors runs without any interaction, leaving the dosimetrists free for other tasks, and minimizes the time spent on the whole process. The complexity of the patient anatomy, the number of loops executed by the algorithm, and the amount of importance factors put on the cost functions will also influence the calculation time.

It should be pointed out that our main purpose is to describe a new and simple method of adjusting importance factors in an automatic way based on prescription value, not for dose comparison with other existing methods because of their intrinsic differences, while in the near future, we will actively carry out the corresponding comparative work to further prove the efficiency of our proposed method. Moreover, different from the other automatic method, our proposed method can be applied not only to dose-based optimization, but also to other optimization models, such as NTCP-based or TCP-based optimization. In other automatic optimization methods, dose-based models were only used.

There persist challenges and room for improvement in this vein. First, the manner in which we adjust the compensation coefficient *k*, mentioned in Optimization Technique section, remains heuristic, due to the uncertain relationship between DVH curves and importance factors. This relationship needs to be further investigated for an efficient way to adjust *k*. Second, while the prostate and HN are partial tumor sites, there is potential for expanding this automatic technique to other tumor sites. Finally, we only study the automatic optimization of weighting factors of the FMO omitting the segmentation of beamlet intensity. In future study, we need to include automatic segmentation of beamlet intensity into the automatic optimization process while keeping the quality of the fluence-optimized dose distribution during segmentation to achieve the real fully automatic optimization. Nevertheless, the proposed method does find importance factors using a reasonably simple method, which was shown in this article. The automatic treatment plan can serve as a reference plan and starting point for the specific treatment and, at least, ensure a certain minimum quality.

Finally, the proposed automatic method can not only be used in inverse treatment planning in IMRT, but can also automatically generate volumetric modulated arc therapy plans. Related work is in progress.

## Abbreviations

CERR: computational environment for radiotherapy research
CI: conformity index
CT: computed tomography
DV: dose–volume
DVH: dose–volume histogram
FMO: fluence map optimization
gEUD: generalized equivalent uniform dose
HI: homogeneity index
HN: head and neck
IMRT: intensity-modulated radiotherapy
NTs: normal tissues
NTCP: normal tissue complication probability
OARs: organs at risk
PTV: planning target volume
TCP: tumor control probability
